# The role of titanium dioxide in enhancing low-emission properties of cementitious materials

**DOI:** 10.1038/s41598-025-06960-4

**Published:** 2025-07-02

**Authors:** Agnieszka Ślosarczyk, Izabela Klapiszewska, Patryk Jędrzejczak, Marta Thomas, Bartosz Gapiński, Marcin Janczarek, Łukasz Klapiszewski

**Affiliations:** 1 Institute of Building Engineering, Faculty of Civil and Transport Engineering, Poznan University of Technology, 60965 Poznan, Poland; 2 Institute of Chemical Technology and Engineering, Faculty of Chemical Technology, Poznan University of Technology, 60965 Poznan, Poland; 3 Institute of Mechanical Technology, Faculty of Mechanical Engineering, Poznan University of Technology, 60965 Poznan, Poland

**Keywords:** Cement composites, Fly Ash, CaCO_3_, Titanium dioxide, Self-cleaning properties, Life cycle assessment, Engineering, Materials science

## Abstract

The need to decarbonize cement binder production and meet the requirements of the circular economy has led to the search for substitutes for cement clinker. Locally available supplementary materials are most commonly used for this purpose. In Europe, these are mainly granulated blast-furnace slag, fly ash and, increasingly, ground limestone. However, the use of multi-component cement binders contributes to differences in strength development characteristics and can affect the performance of functional nanoadditives, including the photocatalytic properties of titanium dioxide. Therefore, this paper investigates the effect of nanometric titanium dioxide (nano-TiO_2_) at 5 wt% on the physico-mechanical, photocatalytic and pro-environmental properties of multi-component cementitious systems. Two-, three- and four-component systems based on Portland cement, fly ash, calcium carbonate and titanium dioxide have been developed, with clinker contents ranging from 35 to 100%. It was shown that nano-TiO_2_ caused an acceleration of the hydration process at the beginning of cement setting, and this effect was enhanced by the presence of a 10 wt% calcium carbonate additive. This had the effect of reducing the porosity of the composites and achieving good mechanical performance. These systems also showed the best phenol degradation efficiency, due to the photocatalytic properties of TiO_2_ enhanced by the presence of calcium carbonate. The presence of fly ash in the systems, at 25 and 50 wt%, slowed down the strength build-up to 90 days of curing, while it resulted in a reduction of the heat of hydration to 200 J/g and a significant reduction of the GWP (Global Warming Potential). Fly ash and calcium carbonate allow the formation of low-carbon cementitious binders and offset the undesirable effect of TiO_2_ on GWP. Unfortunately, large amounts of FA significantly masked the cleaning properties of TiO_2_.

## Introduction

The increased interest in the environmental aspects of cement and cement-based building materials production is also reflected in the number of scientific articles. The resulting relationships between the most frequently used keywords: sustainability development—carbon dioxide—mechanical properties in relation to cement composites were compiled on the basis of records available in the SCOPUS database. The records clearly show a strong correlation between the composition of the cementitious binder and the durability of the cementitious composites, understood in terms of mechanical properties and resistance to external factors. CO_2_ emissions, the environmental assessment of cement production and building materials, and functional composites, e.g. with photocatalytic properties that allow the environment and surfaces to be cleaned of harmful substances, are also frequently discussed.

One material that has been intensively developed in recent years to ensure long-term durability and reusability is nano-engineered concrete. Nano-engineered concrete is an environmentally friendly material, i.e. so-called green concrete, in which cement clinker is replaced by supplementary cementitious materials (SCMs) and which contains nano-additives in its structure that improve its mechanical and durability properties and very often give it new functionalities^1,2^. The most commonly used nano additives in recent times include nano silica, nano-Al_2_O_3_, nano-TiO_2_, nano-Fe_2_O_3_, nano-clay, nano-ZnO and carbon-based materials such as carbon nanotubes or graphene oxide^3–8^. The presence of nanoparticles capable of reacting with the cement matrix, such as nano-silica, causes a series of positive reactions during the setting of the cement binder, such as the acceleration of the hydration process, refinement of the microstructure of the binder as a result of nucleation and filling effects, which consequently contribute to an increase in the strength of the cement composite and an improvement in its long-term durability in the face of many external factors, such as carbonization, chlorides or the effects of increased or decreased temperatures. Nano-additives also very often add new functionalities to cementitious composites; in this respect, cementitious composites with self-cleaning (nano-TiO_2_), antimicrobial (nano-ZnO), self-healing (encapsulation of healing agents or bacteria) or self-sensing (carbon nanotubes—CNTs, graphene oxide—GO) properties have been developed in recent years^9–12^.

Among nano-engineered cementitious composites with self-cleaning properties, titanium dioxide is the most commonly used due to its stability in the cement matrix and good photocatalytic properties. It is typically added at levels of up to 5% by weight of the cement, either as a protective coating or as an admixture to the cement binder. Recently, particular attention has been paid to the compatibility of admixtures with low-emission cementitious binders containing high amounts of SCMs, especially in the case of cement composites modified with nanometric titanium dioxide, whose effective photocatalytic activity depends on a number of factors, the most important of which are the size of the nanoparticle, its specific surface area and roughness, and its crystalline form^13–15^. The study also showed a particularly beneficial effect of titanium dioxide ground with FA, resulting in an increase of more than 30% in early flexural and compressive strength^16–19^. Other researchers have indicated that titanium dioxide contained in the titanium slag (TS) used with fly ash (FA) to make the cement composite can reduce weight loss by almost 20% and reduce strength loss after 800 ℃ heating by almost 32%, concluding that titanium dioxide has a positive effect on the durability of the low-emission cement composite^20^. However, the production of titanium metal, a precursor for TiO_2_, is both costly and technically demanding^21^. Moreover, the most common methods for obtaining titanium dioxide—namely the sulfate and chloride processes – pose significant environmental challenges^22^. Considering the high production costs, environmental concerns, and the growing demand that may outpace supply capabilities, there is a clear need for the prudent use of TiO_2_^23^. This also underscores the importance of exploring alternative materials that can mitigate its limitations. The solution is to use of FA in the production of cementitious composites, is undoubtedly a green solution, allowing the incorporation of post-industrial waste into a new building^24,25^ which results in less waste going to landfill. However, it is necessary to take into account the predictions of declining FA production especially in North America, Eastern Europe or Australia, and the increase in FA production in China or India, which may result in the need to transport over long distances^26^. Most of the research work has focused on these aspects, while there are few studies that include aspects of the dependence of the photocatalytic properties of nano-TiO_2_ and low-emission cementitious binders containing large amounts of SCMs. The issue of oxide compatibility with low-emission mixtures, which, in addition to the introduced TiO_2_ and oxides typical of cement clinker, contain numerous other oxides, is highly significant. This is particularly important because the presence of a large number of such compounds may interact in less favorable ways, for instance, by hindering setting processes, causing reductions in strength and in workability/flowability, and the tendency of NPs to agglomerate with one another in the cement matrix^27,28^.

The use of calcium carbonate is becoming increasingly important in the many studies currently being conducted on low-emission composites. The growing interest in this material has led to the development of blended cements such as Portland-Limestone Cement (PLC), in which limestone is used as a filler, reducing the need for clinker. In California and the USA, PLC typically consists of about 5% gypsum and 5–15% ground limestone, and its consumption in the USA in 2022 amounted to over 90% of the blended cements used, representing a group of 24% of the total market. Low levels of clinker conversion to limestone in PLC have little effect on the properties of the material. It is worth mentioning here the work on LC3 technology (limestone calcined clay cement)—a ternary cement in which 50% of the composition is clinker. By exploiting the wide availability of limestones and clays, LC3 can be used on a large scale^29^.

Therefore, in the present study, low-emission cementitious binders were developed containing different amounts of fly ash and calcium carbonate at the expense of cement clinker, whose content ranged from 35 to 100%, and the effect of nanometric titanium dioxide on the physico-mechanical and photocatalytic properties of the prepared systems was studied. The evaluation of the mechanism of action was carried out by measuring the heat of hydration, mechanical and structural characterization by means of computed microtomography and scanning electron microscopy, while the cleaning properties were studied by using phenol degradation test.

## Experimental

### Materials

To produce low emission cement composites doped with titanium dioxide, the following were used: Portland cement CEM I 42.5R (Górażdże Cement S.A., Górażdże, Poland), standard quartz sand with φ < 2 mm (Kwarcmix, Tomaszów Mazowiecki, Poland), fly ash (Opole Plant, Opole, Poland), titanium dioxide (TiO_2_) in anatase form – nanopowder; 99.7% trace metals basis; BET surface area: 45–55 m^2^/g; density: 3.9 g/mL at 25 °C; CAS number 1317-70-0 (Sigma-Aldrich, Merck, Darmstadt, Germany), calcium carbonate (CHEMPUR, Piekary Śląskie, Poland) and distilled water.

**Fig. 1:**
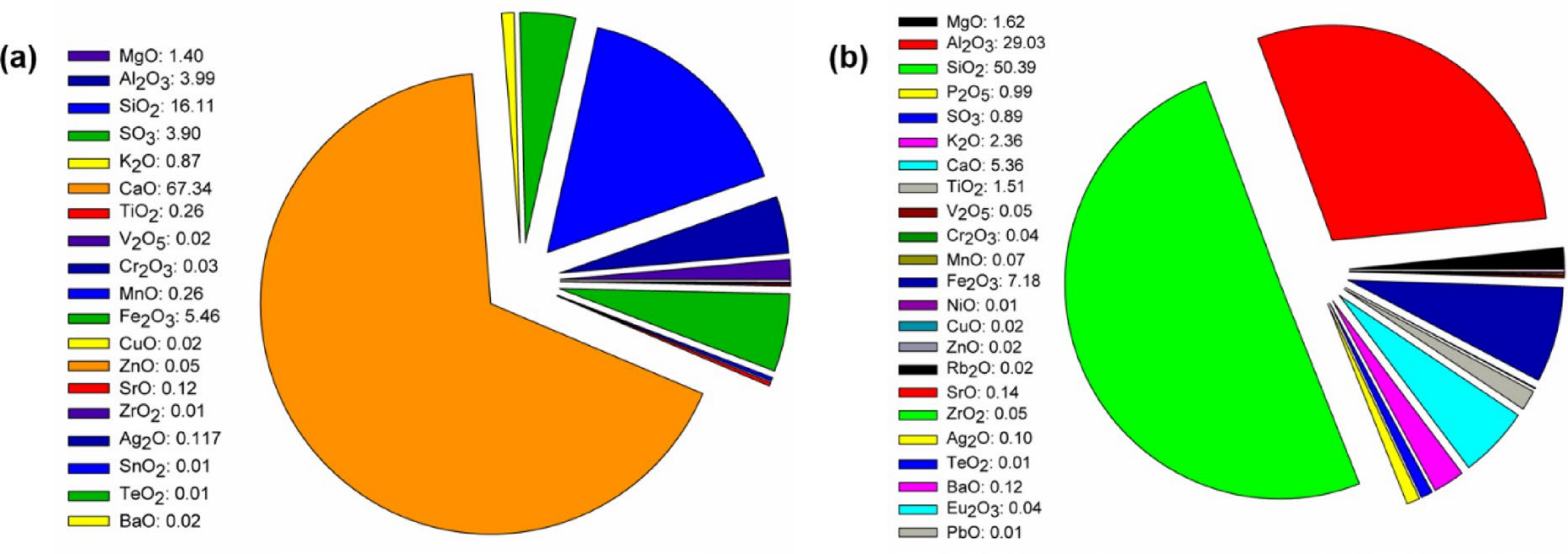
The oxide composition of (**a**) cement clinker and (**b**) FA, data presented in (%).

The Portland cement used in the tests, according to the manufacturer’s specifications, consists of Portland clinker, calcium sulfate (gypsum), dust from exhaust gases (from cement production), and ferrous sulfate, the weight concentrations of which in the cement are respectively: 86.5–100%; 0–8%; 0–5% and 0–1%. The detailed oxide composition is presented in Fig. 1a. The calcium carbonate (CC) used, with a molecular weight of 100.09 g/mol, is a white, odorless solid with a relative density (20 °C) of 2.7 g/cm^3^. The fly ash (FA), which is added to the mixtures in the form of a dry gray solid, contains a number of oxides (in descending order of content): SiO_2_ (50.39%), Al_2_O_3_ (29.03%), Fe_2_O_3_ (7.18%), CaO (5.36%), K_2_O (2.36%), MgO (1.62%), TiO_2_ (1.51%). It also contains small amounts (less than 1%) of oxides such as P_2_O_5_, SO_3_, and ZnO. The full oxide composition of FA is presented in Fig. 1b, and additionally, the particle size distribution of the utilized powder materials is provided in Table 1.

**Table 1:** Particle size distribution of the utilized powder materials.

Powder material	Particle size distribution (nm)
Cement clinker	190–615; 955–1718
FA	122–255; 531–3091
TiO_2_	< 25 (data provided by the manufacturer)
CC	255–1106

### Preparation of cement composites

**Fig. 2:**
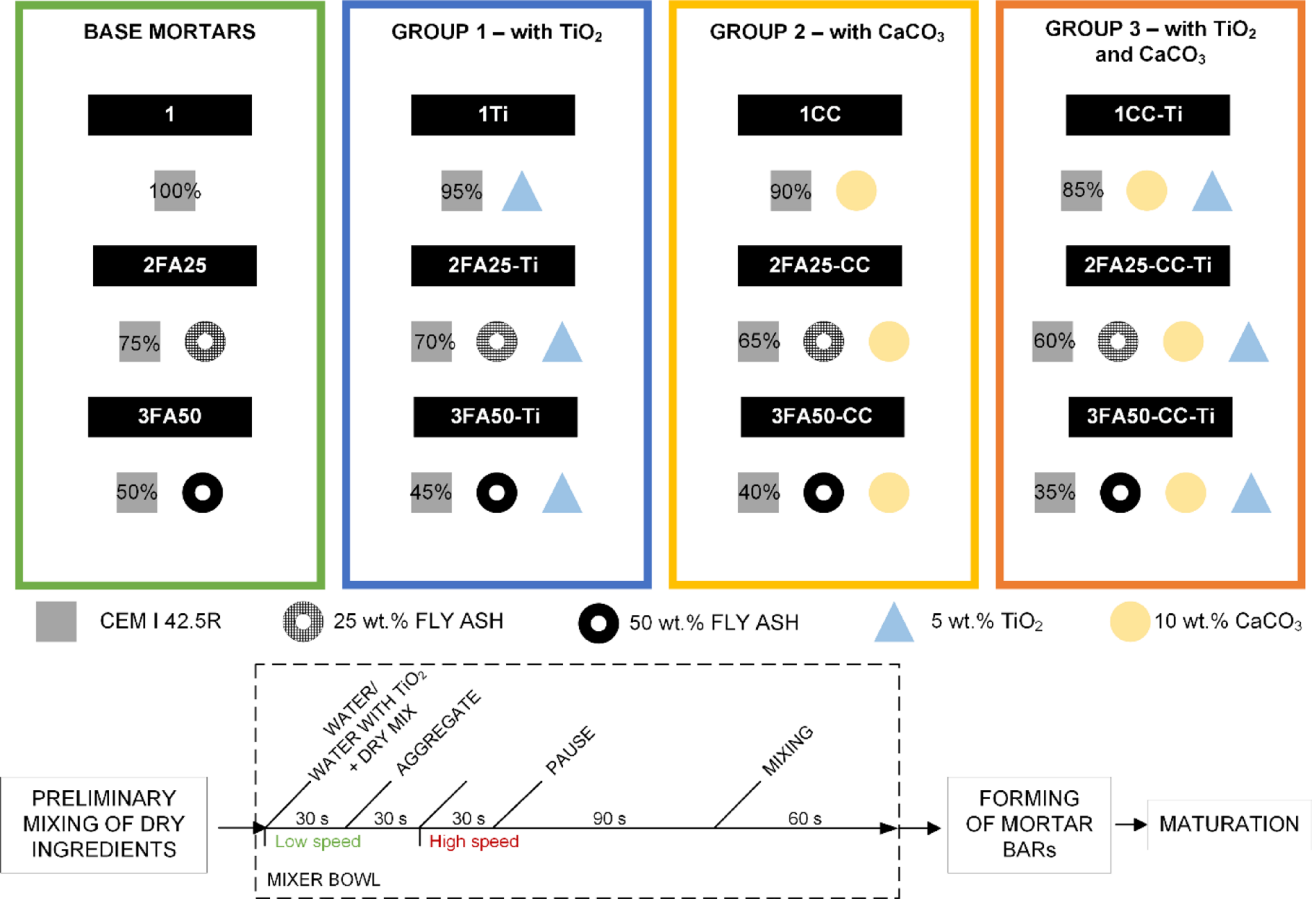
Schematic composition of the mixes and the whole procedure.

The fresh mortar was prepared in accordance with PN-EN 196-1 using an automatic mortar mixer. The base consists of three cement matrices (marked as 1, 2FA25 and 3FA50) consisting of cement, aggregate, water and 0%, 25% and 50% fly ash, respectively. Composites with Ti are characterized by the addition of 5% TiO_2_, while those of CC are characterized by the addition of 10% calcium carbonate. The weight of the components of the mixture was constant, the addition of fly ash, calcium carbonate or TiO_2_ was associated with a reduction in the weight of the added cement. The components of the mixture, i.e. water, binder and quartz sand, were each in a ratio of 1:2:3, the detailed composition of the binder is shown in Fig. 2 as well as the whole procedure. The added fly ash and/or calcium carbonate was first dry mixed with the cement, and TiO_2_ was introduced as a suspension prepared with a magnetic stirrer in the working water.

### Characterization of cement composites

The analyses and tests performed on the fresh and the hardened mixes are summarized in Table 2.

**Table 2:** List of tests and analyses performed on the discussed cement composites.

Test	Short description, standard	Equipment
Consistency of fresh mortar	Flow table test based on EN 1015-3	Shaking table
Heat of hydration	Measurement of the amount of heat that is released during the setting process based on EN 196-9	Semi-adiabatic calorimeter (Testing, Berlin, Germany)
Morphology	Microphotographs obtained with a scanning electron microscope	Scanning electron microscope (Tescan Orsay Holding a.s., Brno, Czech Republic)
Non-invasive computed tomography (CT)	X-ray radiation microtomograph with nanofocus lamp (voltage of 180 kV and a power of 15 W) and microfocus lamp (voltage of 240 kV and a power up to 320 W)	Phoenix V|tome|x S240 microtomograph (GE Waygate Technologies, Hürth, Germany)
Mechanical properties	Flexural and compressive strength tests based on EN 196-1	Matest Servoplus Evolution strength machine (MATEST S.p.A., Treviolo, Italy)

### Photocatalytic activity

The 40 mm x 40 mm x 5 mm tiles, cut from beams prepared for bending and compression strength tests, were used to determine the photocatalytic properties of cement composites. Quartz tubes with an inner diameter of 32 mm, a wall thickness of 2 mm, and a height of 70 mm were affixed to these tiles using silicone sealant. The prepared systems allowed for achieving consistent dimensions of analyzed surface during the test. The assessment of photocatalytic properties involves analyzing the concentration of a model organic pollutant during the test. In this study, a 10 mg/L solution of phenol was chosen for this purpose. For the quantitative assessment of phenol, a colorimetric method was employed, which involve the coupling of phenol with 4-nitroaniline diazonium chloride. For this purpose, a 0.005 M solution of 4-nitroaniline, a 0.5 M solution of sodium carbonate, and a saturated solution of sodium nitrate(III) were prepared. A detailed description of solution preparation has been presented in our previous work^15^.

**Fig. 3:**
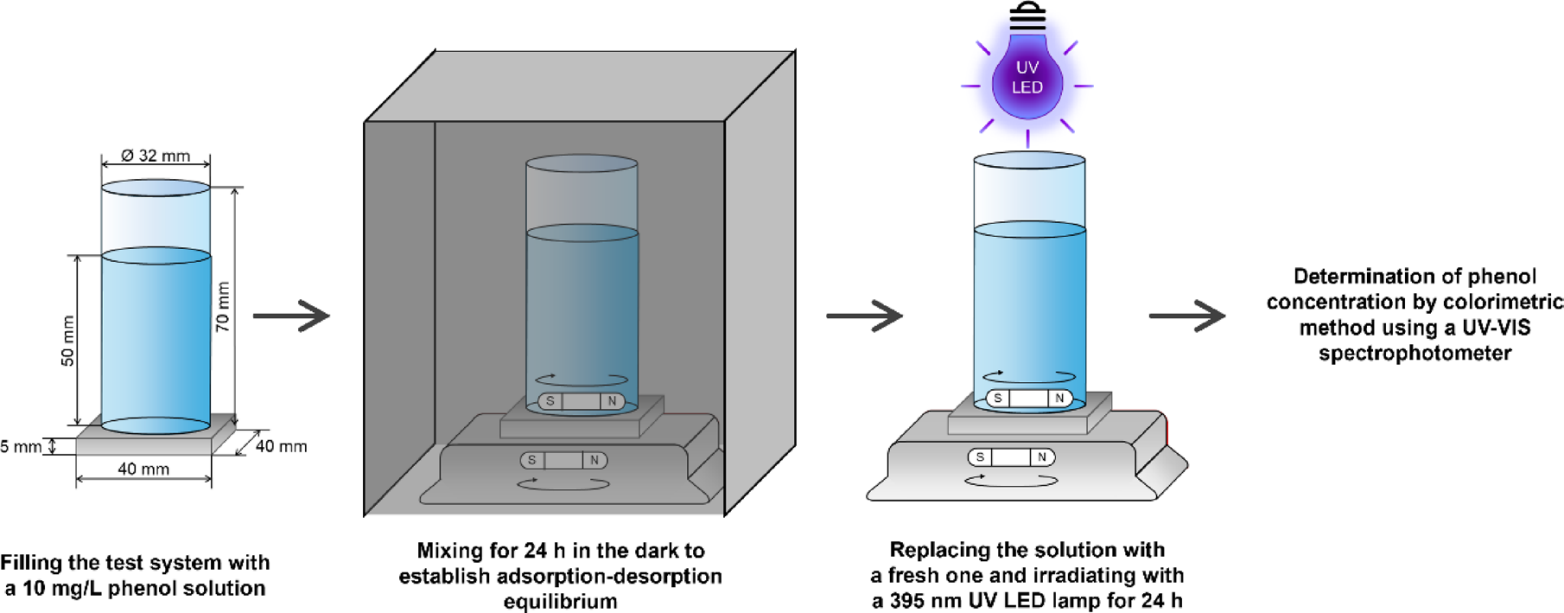
Schematic diagram of the photocatalytic activity test of the obtained cement composites.

An LED (Light Emitting Diode) lamp from Bridgelux (Fremont, California, USA) was used to evaluate the photocatalytic properties of cement composites. It is characterized by ultraviolet radiation (wavelength 395 nm), a power of 50 W, and a cooling system equipped with a radiator and a fan. Its construction using chip-on-board technology allowed the dissipation of the heat generated during the irradiation of the samples, ensuring stable temperature conditions. The intensity of ultraviolet radiation was 500 ± 10 mW/cm^2^. Before assessing the photocatalytic properties of cement composites, it was necessary to establish the adsorption-desorption equilibrium. To achieve this, 40 mL of a 10 mg/L phenol solution was introduced into the system and stirred for 24 h in the absence of light. In the next stage, the solution was replaced with a new one of the same concentration, and after 30 min of stirring in the dark room, the change in phenol concentration was checked. At this point, the LED lamp was turned on, and 5 mL of subsequent samples were collected after 6 and 24 h of exposure. Figure 3 presents the schematic diagram of the photocatalytic activity test. To remove solid impurities from the solutions, they were passed through syringe filters (Macherey-Nagel, Duren, Germany). Subsequently, 5 mL of the previously prepared base solution of 4-nitroaniline diazonium chloride was added to the 5 mL sample, and the mixture was shaken for approximately 1 min. The prepared samples were left for 3 h, after which the concentration of phenol was determined. For this purpose, the absorbance of the solution was measured at a wavelength of 477 nm using a UV-VIS spectrophotometer (V-750, Jasco, Tokyo, Japan) to obtain spectra, and the concentration of phenol was determined from the standard curve.

Finally, the degradation efficiency of phenol, $\:Q$, was calculated using the formula:

$$\:Q=\frac{{C}_{p}-{C}_{k}}{{C}_{p}}*100\%$$

where $\:{C}_{p}$ - initial phenol concentration, namely 10 mg/L, $\:{C}_{k}$ - phenol concentration read from the standard curve after 6–24 h of exposure.

### Life cycle assessment

**Fig. 4:**
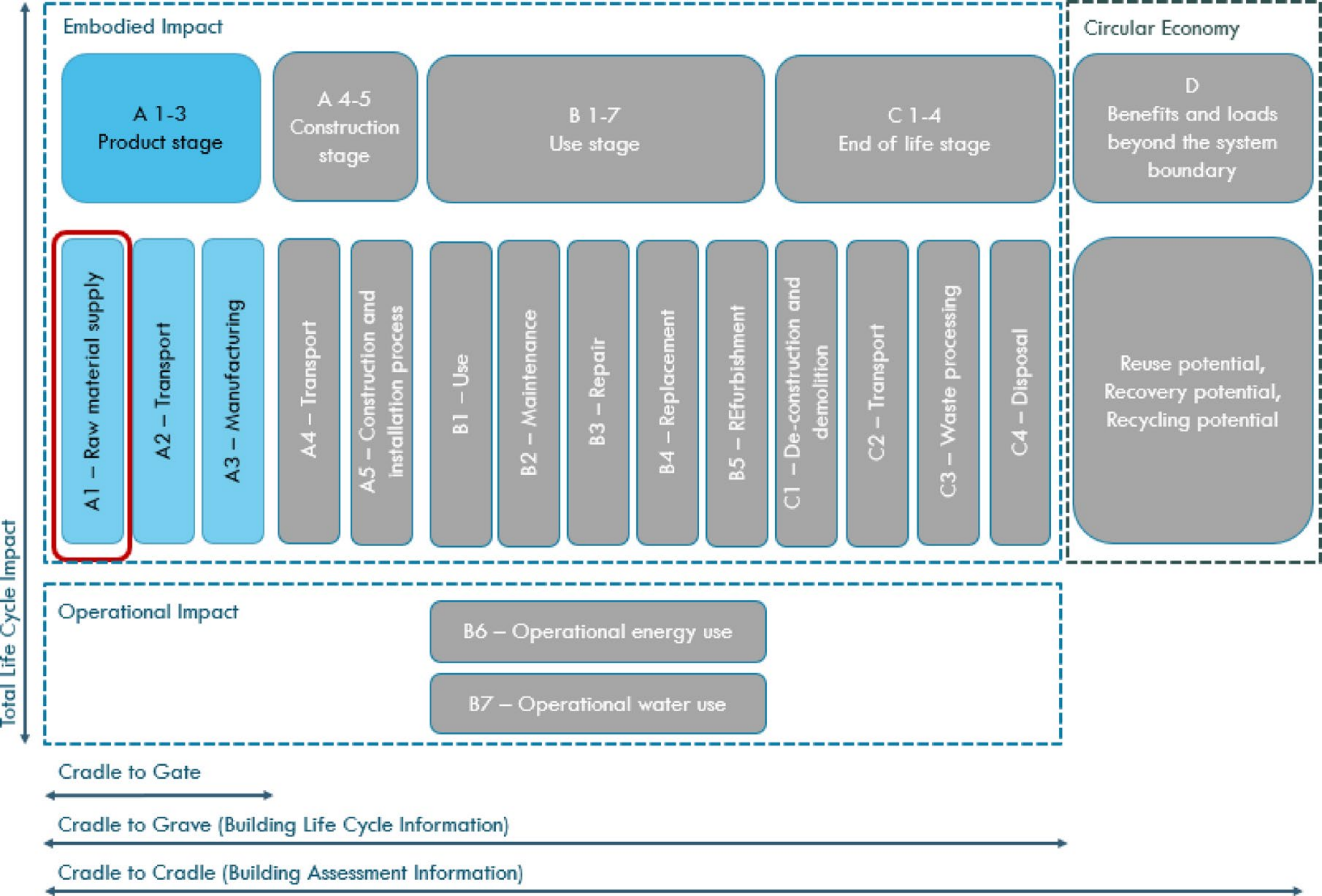
Life cycle stages according to EN 15804.

An environmental life cycle assessment (LCA) of the mortars was carried out for their environmental impact by calculating the emissivity of the different mixes. The life cycle assessment is a comprehensive tool that enables the tracking of environmental impacts at each stage of a product’s life cycle. The LCA analysis may include indicators such as: GWP (Global Warming Potential)—climate change associated with greenhouse gas emissions, AP (Acidification Potential)—acidification, EP (Eutrophication Potential)—eutrophication, ODP (Ozone Depletion Potential)—ozone layer depletion, POCP (Photochemical Ozone Creation Potential)—photochemical smog formation. The life cycle of a product is typically divided into the product stage, construction stage, use stage, end-of-life stage and benefits beyond the system. The division into life cycle stages according to the EN 15804 standard is presented in Fig. 4. For the mortars studied, the calculations were carried out based solely on the GWP of individual raw materials, i.e. they only considered the product stage limited to module A1 of EN 15804. It was decided not to include modules A3 (manufacturing) and A2 (transport) because of the comparability of these values across all blends and the small impact of A2 and A3 on the global warming potential associated with the cradle-to-gate production stage. The authors made this choice because the A1 stage is the most difficult to reduce the amount of carbon dioxide emitted. For each type of material used, global warming potential data was extracted from information such as Environmental Product Declarations (EPDs) and OneClickLCA software.

## Results and discussion

### Heat of hydration

**Fig. 5:**
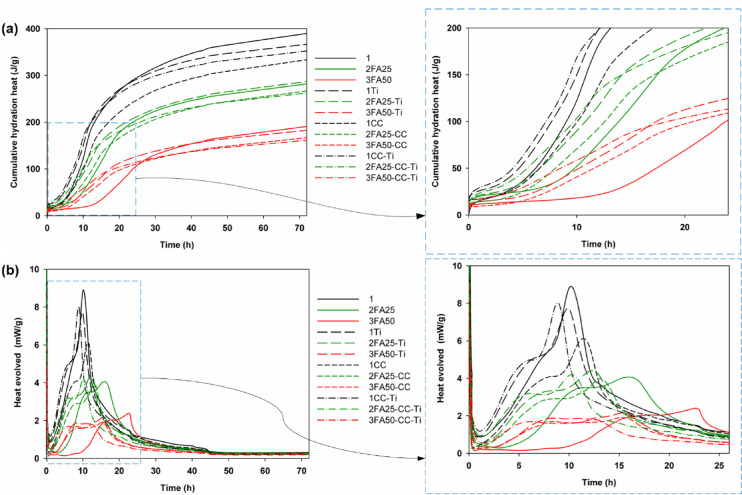
Heat of hydration results for all analyzed samples presented as (**a**) cumulative hydration heat and (**b**) heat evolved over time.

Figure 5 and Table 3 shows the results of the heat of hydration of the produced systems, recorded during the first 72 h of binder setting. The results were analyzed in 3 groups according to the fly ash content. It was shown that the substitution of 25 and 50% fly ash in the cement clinker significantly reduced the heat of hydration released. Analyzing 1 group of systems on pure clinker cement (samples 1, 1Ti, 1CC and 1CC-Ti), it can be seen that the addition of TiO_2_ at 5% by weight of cement (1Ti) accelerates the hydration process of the cement binder. This is in agreement with the results of other researchers, nano-TiO_2_ does not have binding properties like nano silica, but due to its nanometric dimensions it provides additional active centers from which the growth of the C-S-H (calcium silicate hydrate) phase starts, and this effect is called nucleation^28,30–32^. When the cement clinker was replaced with 10% calcium carbonate (1CC sample), the opposite effect was obtained; a significant reduction in the heat evolved was observed, which may be related to the so-called dilution effect. Like nano-TiO_2_, calcium carbonate has no binding properties and its effect is purely physical, related to the filling effect. Nevertheless, a positive effect on the hydration time was observed for the combined use of nano-TiO_2_ and calcium carbonate (1CC-Ti sample). In this case, an increase in hydration heat evolved was observed, which was higher than when TiO_2_ was used alone (1Ti sample). An increase in the cumulative heat of hydration for calcium carbonate has already been observed in the literature, e.g. in the work of Wu et al.^33^ nano-CaCO_3_ up to 3.2 wt% resulted in a reduction of the resting time from 13 to 9 h. This was attributed to a nucleation effect. Similar relationships were observed in the other two groups where cement clinker was replaced by fly ash, with all the multi-component systems having a lower cumulative heat of hydration after 72 h of setting compared to pure cement mixes. The three and four component systems had a low heat of hydration of less than 270 J/g for mixes with 25% FA (2FA25, 2FA25-Ti, 2FA25-CC and aFA25-CC-Ti samples) substitution and less than 200 J/g for 50% FA (3FA50, 3FA50-Ti, 3FA50-CC and 3FA50-CC-Ti samples respectively), making them low emission binders.

It is worth noting that FA-containing cement composites are also characterized by the longest initial setting times, as shown in Table 3. The addition of TiO_2_ or CaCO_3_ to the system enables a reduction in the initial setting time by approximately 7 h, while the combined effect of these two additives results in a cement composite with an initial setting time comparable to that of typical clinker-based cement mortar. A similarly beneficial effect was observed when analyzing the data related to the final setting time. For the 3FA50-CC-Ti composite, the final setting time was approximately 12 h, representing a 10-hour acceleration in the completion of hydration processes compared to the 3FA50 sample. The combination of limestone with FA in cementitious composites has already been reported in the literature^34^ indicating properties that accelerate setting. The authors of that study compared the effects of nano- and micro-limestone, confirming the beneficial influence of even small amounts of micro-limestone. It is important to emphasize that the shortened setting time did not negatively affect long-term strength, and, due to the wide availability of limestone in the USA, it can be successfully utilized in cement mortars.

Regarding studies on the heat of hydration of composites doped solely with TiO_2_, Chen et al.^30^. conducted research on cement pastes with 5% and 10% TiO_2_ admixtures. They demonstrated that TiO_2_ accelerated hydration processes, which is consistent with the findings of the present study, while not causing an increase in the heat evolution rate during hydration, but rather contributing to an overall increase in cumulative heat release.

The solution proposed in this study—combining the action of CaCO_3_ and TiO_2_ in cement composites containing up to 50% fly ash—is an innovative approach that can be successfully applied in the technology of modern binders, thus addressing the demands of sustainable construction and supporting the goals of decarbonization and carbon footprint reduction programs.

**Table 3:** Initial and final setting times of all tested mortars.

Sample	Initial setting time (h)	Final setting time (h)
1	~ 2.5	~ 10.0
2FA25	~ 5.5	~ 16.0
3FA50	~ 10.5	~ 22.0
1Ti	~ 1.5	~ 10.0
2FA25-Ti	~ 2.3	~ 12.0
3FA50-Ti	~ 3.2	~ 15.0
1CC	~ 2.0	~ 11.0
2FA25-CC	~ 2.0	~ 13.0
3FA50-CC	~ 3.0	~ 15.0
1CC-Ti	~ 2.0	~ 9.0
2FA25-CC-Ti	~ 2.0	~ 10.0
3FA50-CC-Ti	~ 2.5	~ 12.0

### Physico-mechanical and structural characterization of cement composites

**Fig. 6:**
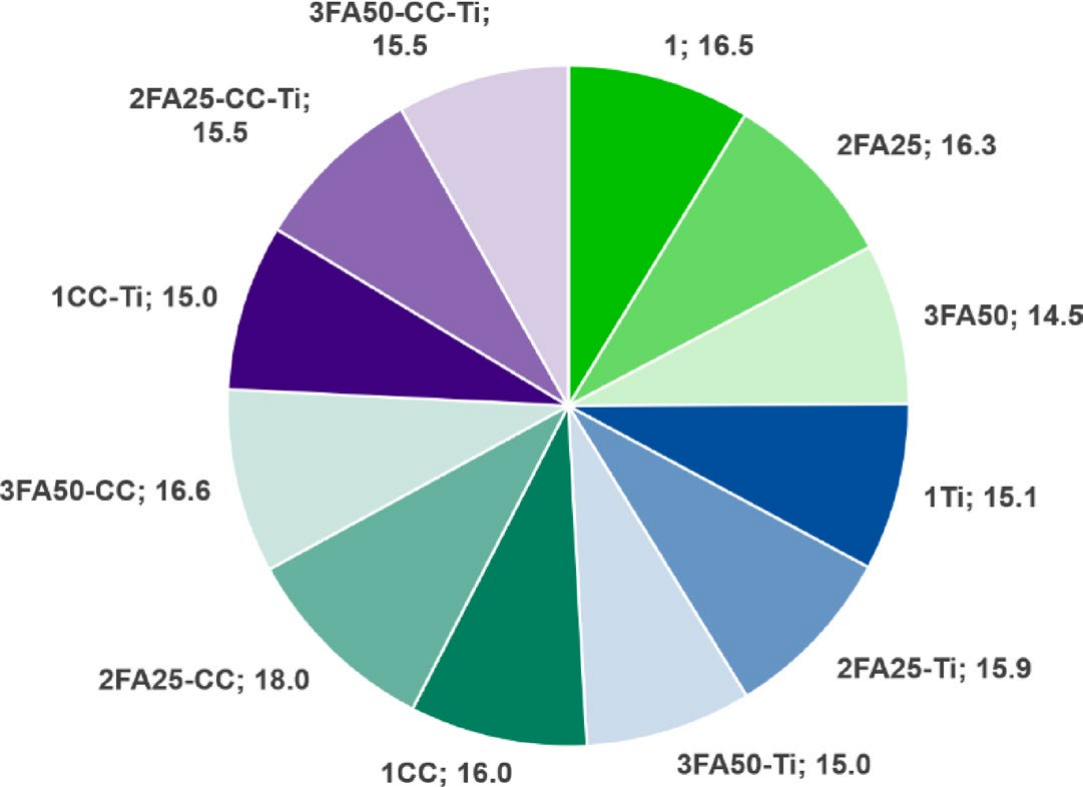
Slump test results for all fresh mortar samples (all data presented in cm).

**Fig. 7:**
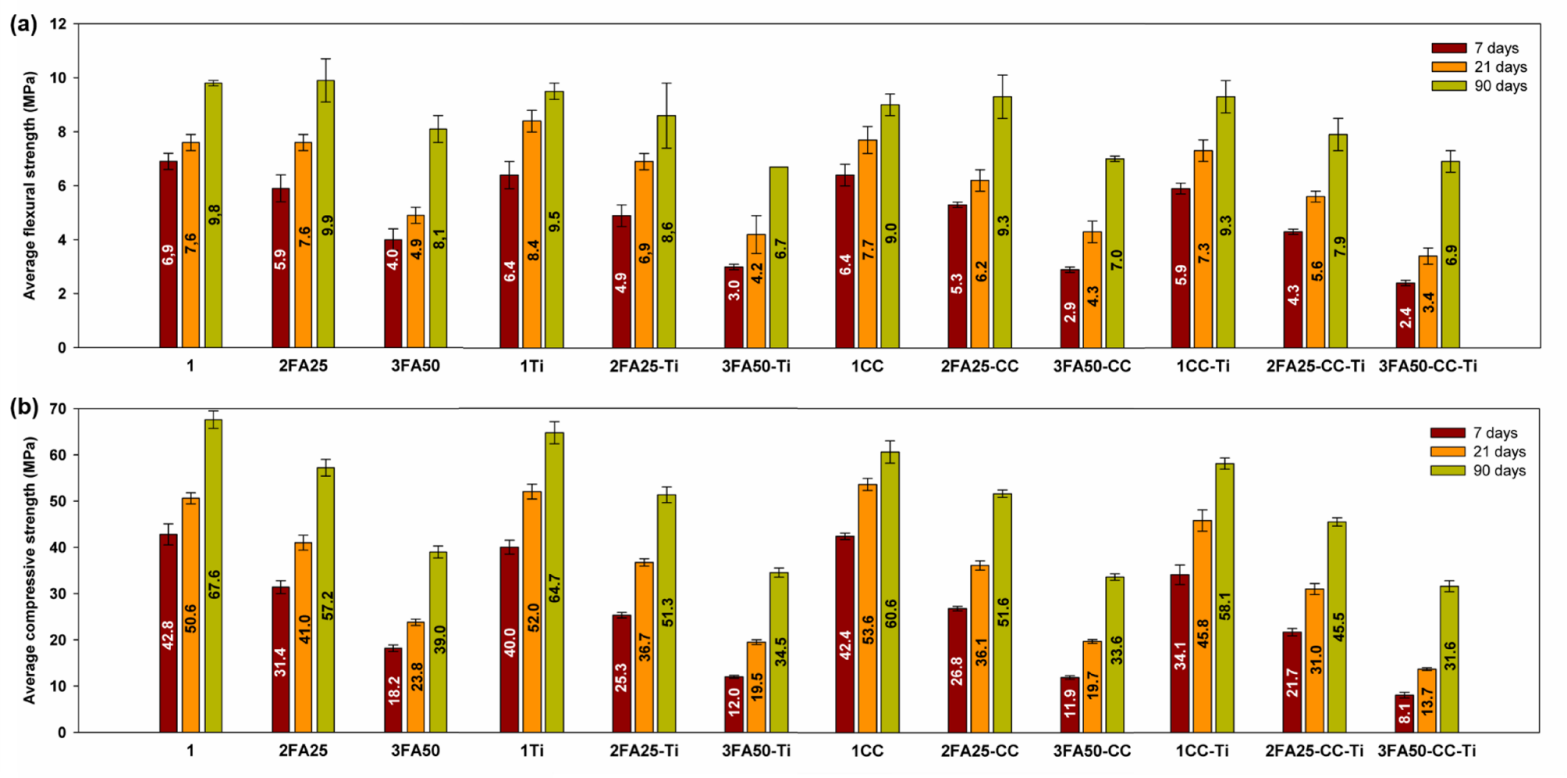
Average (**a**) flexural and (**b**) compressive strength results for all analyzed mortar systems.

The results for slump, density, flexural and compressive strength and the total voids volume for the mixtures tested are shown in Table 4; Figs. 6 and 7.

Analysis of the slump results on the shaking table (see Fig. 6) showed that the addition of fly ash at 25% (2FA25 sample) did not affect the workability of the mortars, only at 50% a reduction (3FA50 sample) in the spread diameter of the mortar from 16.5 to 14.5 cm was observed. Similarly, the addition of nano-TiO_2_ at 5% (1Ti sample) reduced the workability, whereas the presence of FA in the all mixes slightly improved the workability (2FA25-Ti sample). The addition of 10% calcium carbonate alone (1CC sample) did not reduce the flowability of the mixes, while a negative synergistic effect on consistency was observed when used with nano-TiO_2_ (1CC-Ti). A significant improvement in flowability to a value of 18 cm was observed for a ternary systems containing 25% FA and 10% CaCO_3_ (2FA25-CC) and to 16.6 cm for 50% FA and 10% CaCO_3_ (3FA50-CC) in addition to cement clinker. A negative effect on slump flow for nano-TiO_2_ was also observed by Joshaghani et al.^35^. They estimated an acceptable value of TiO_2_ in concrete mixtures at 3%, while a decrease in workability of self-compacting concretes was observed at 5%. They attributed this to the high surface area of TiO_2_, which tends to absorb water and reduce slump flow. Similarly, research by Shaikh and Supit^36^ on nano-CaCO_3_ showed a reduction in slump values from 140 mm to 135 and 120 mm for mixtures containing 0, 1 and 2% nano-additive respectively.

Analysis of the flexural and compressive strength results (see Fig. 7) was carried out at the early binder setting times of 7 and 21 days and at the longer time after 90 days to assess the strength development characteristics, particularly for mixes with low cement clinker content. It was shown that for mixes containing pure cement (1 sample), TiO_2_ (1Ti sample) and calcium carbonate (1CC sample), an improvement in compressive strength of several percent was obtained, with the improvement disappearing after 90 days of setting. Otherwise, a decrease in both flexural and compressive strength, related to the dilution effect, was observed with increasing fly ash content in two-, three- and four-component systems. Unfortunately, the combined addition of nano-TiO_2_ and calcium carbonate in systems containing FA (2FA25-CC-Ti and 3FA50-CC-Ti samples) was not able to accelerate the hydration process of the cementitious binder sufficiently to produce significant increases in flexural and compressive strength. Nevertheless, interesting results were obtained for the ternary systems 2FA25Ti and 3FA25CC, for which the flexural and compressive strengths reached high values close to 9 and 51 MPa, respectively, and were slightly lower than those obtained for pure cement clinker (1 sample). The four-component systems showed the lowest strength gains, while the results obtained after 90 days of curing reached relatively high values of 7.9 and 45.5 MPa for 2FA25-CC-Ti system and 6.9 and 31.6 MPa for 3FA50-CC-Ti sample.

**Fig. 8:**
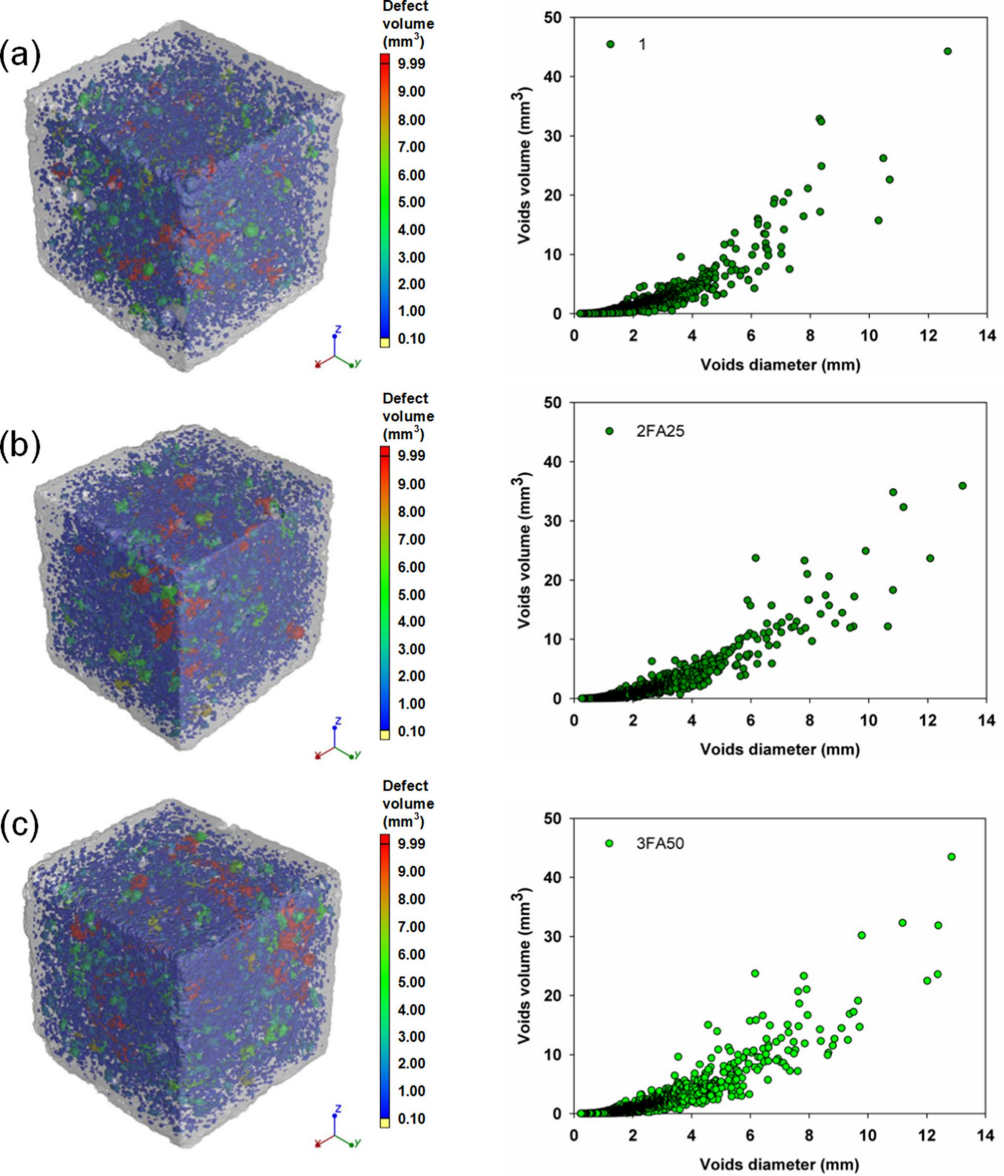

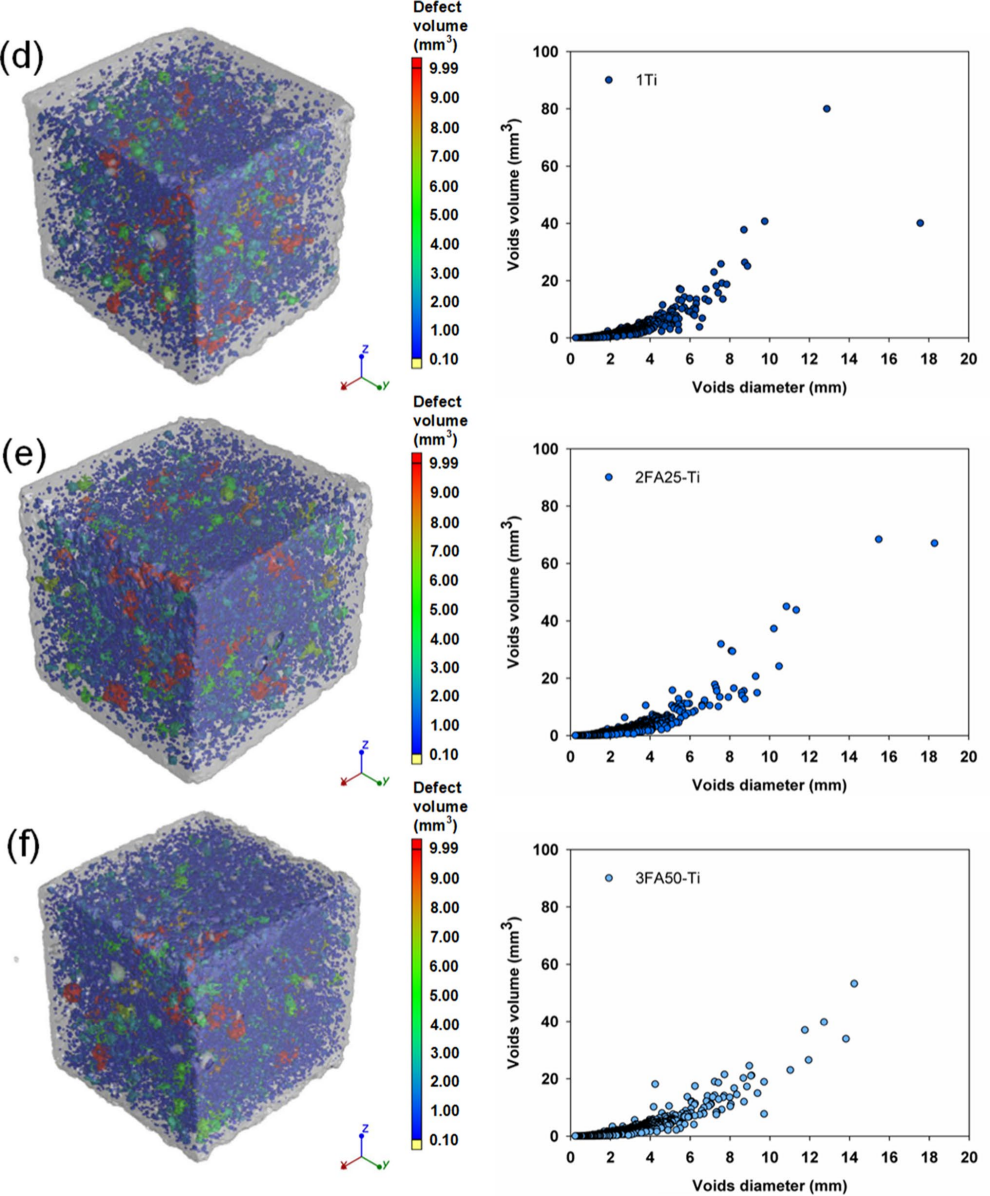

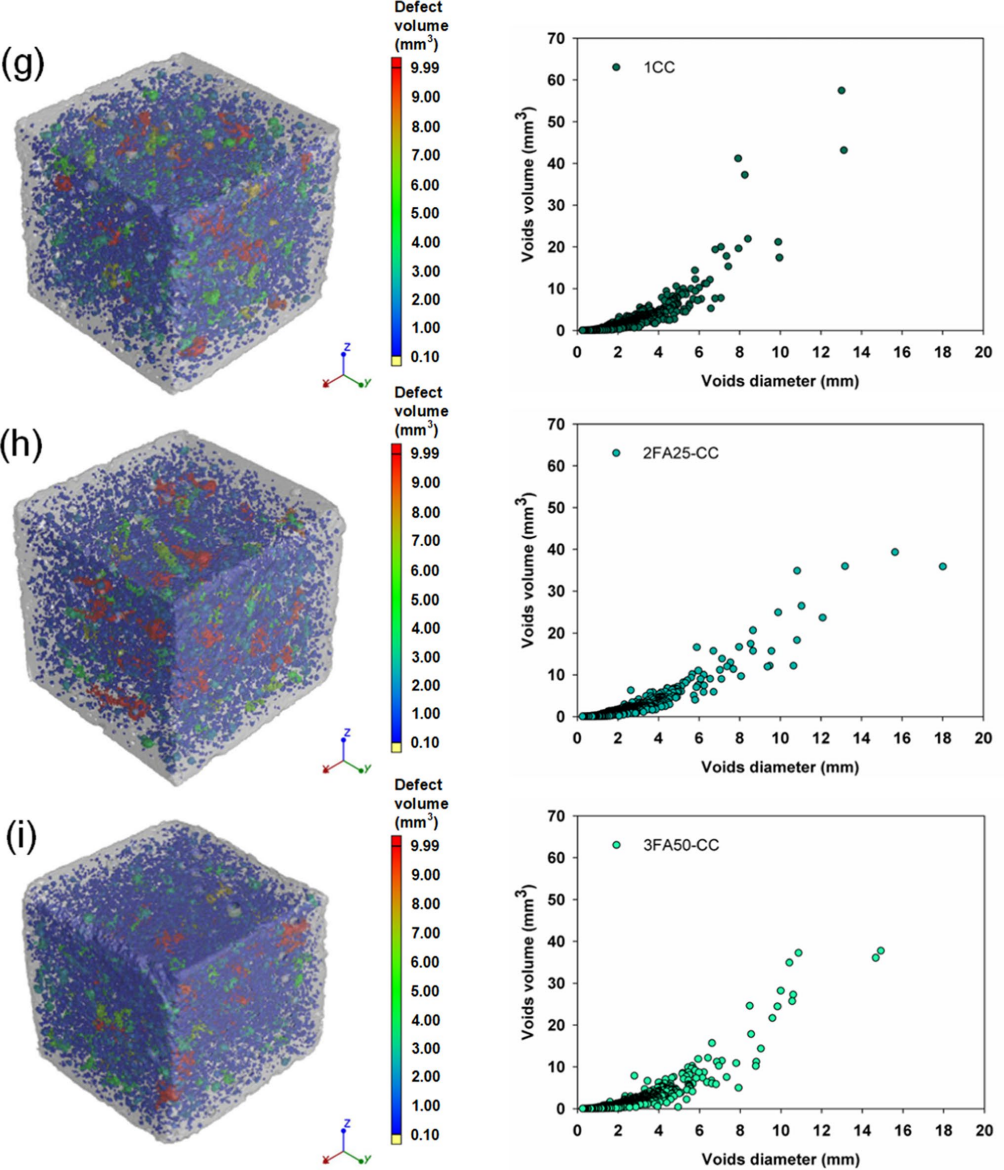

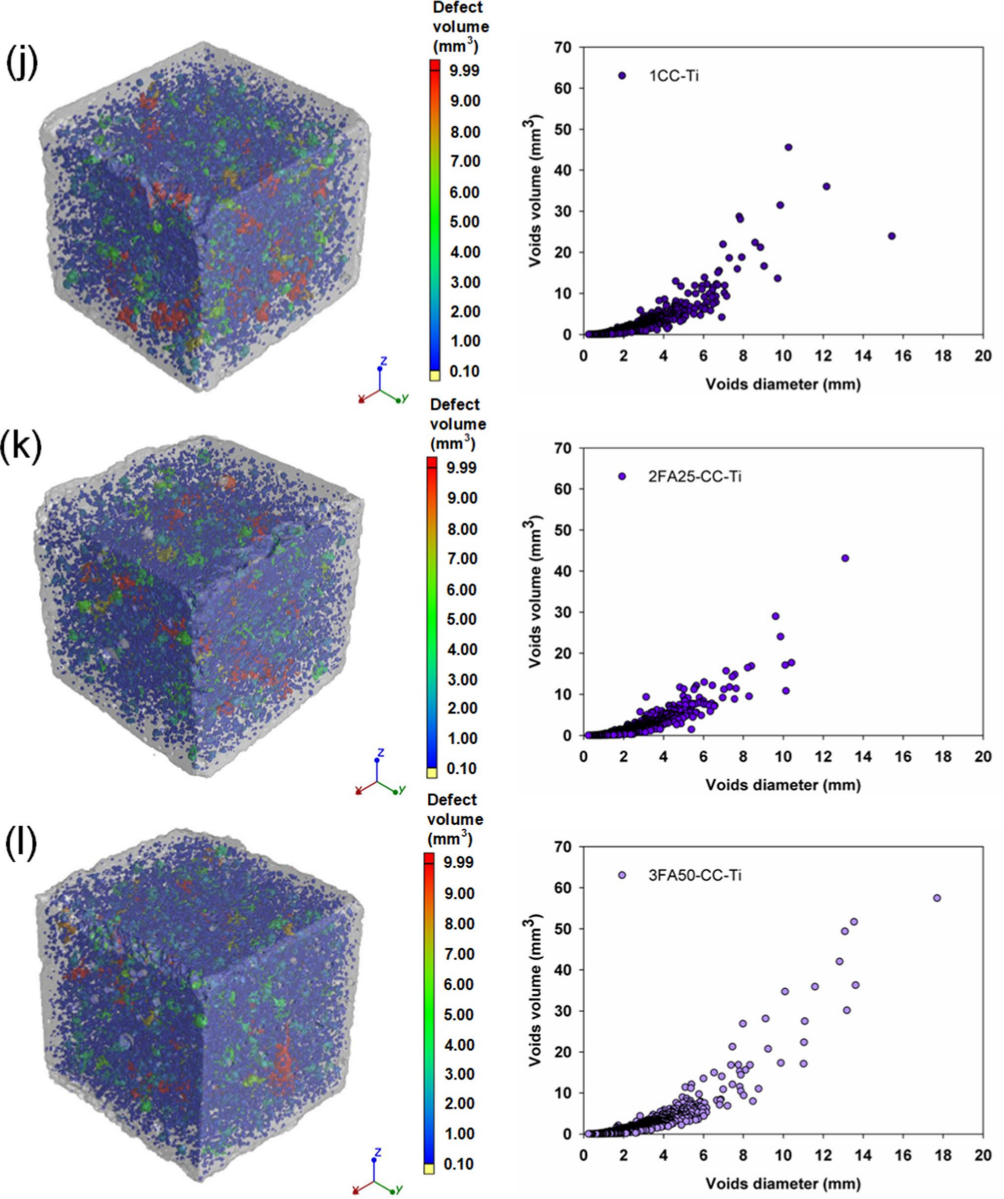
Images of samples with the presented pore distribution in terms of their diameter and volume for (**a**) 1, (**b**) 2FA25, (**c**) 3FA50, (**d**) 1Ti, (**e**) 2FA25-Ti, (**f**) 3FA50-Ti, (**g**) 1CC, (**h**) 2FA25-CC, (**i**) 3FA50-CC, (**j**) 1CC-Ti, (**k**) 2FA25-CC-Ti and (**l**) 3FA50-CC-Ti composites.

**Fig. 9:**
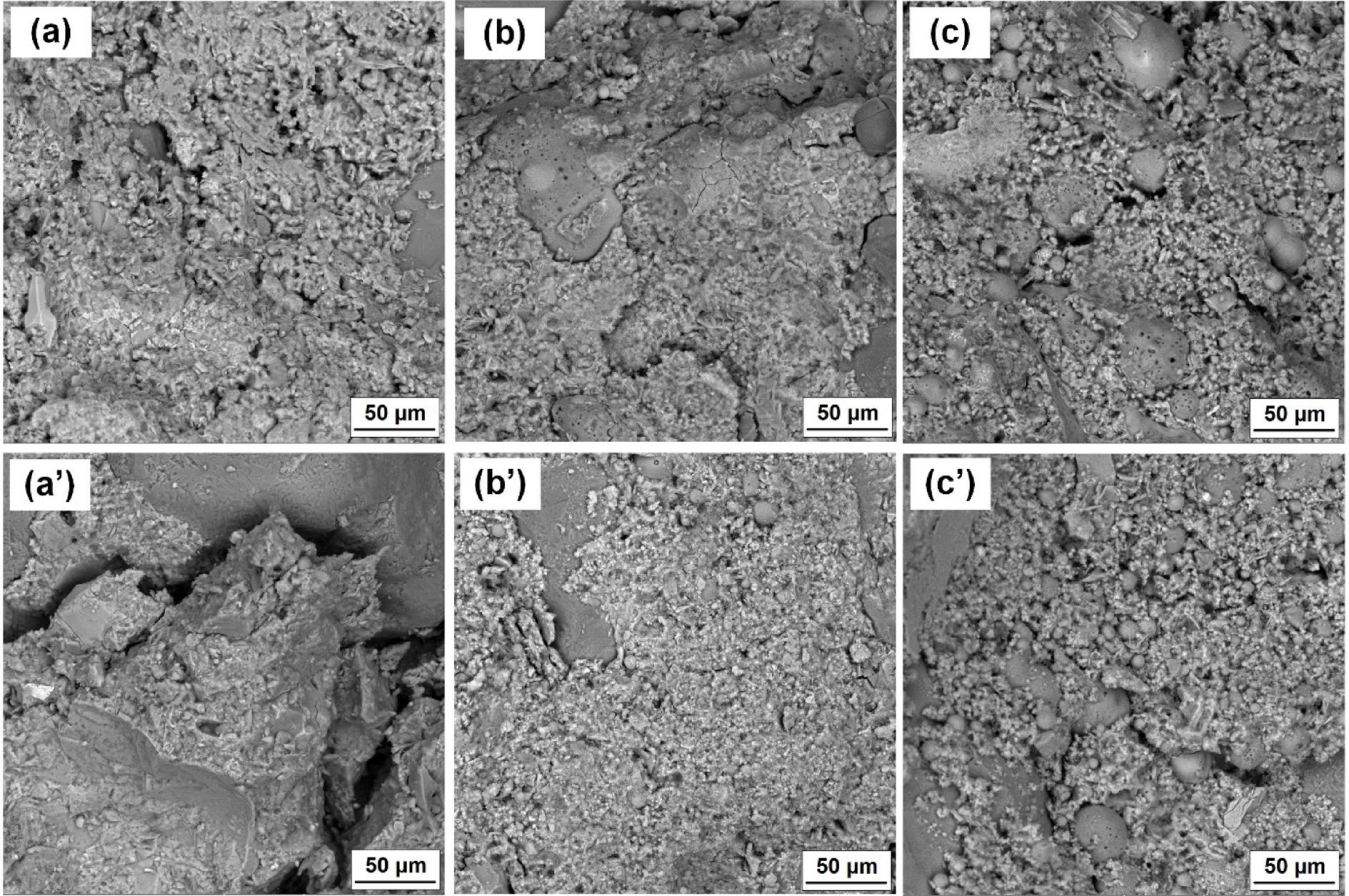
Scanning electron microscopy (SEM) images for samples: (**a**) 1, (a’) 1Ti; (**b**) 2FA25, (b’) 2FA25Ti; (**c**) 3FA50, (c’) 3FA50Ti, respectively.

The densities measured were typical of cement mortars, ranging from 2.15 to 2.34 g/cm^3^ and reflecting the hydration progress of the systems tested. However, a trend was observed: the composites with 25 and 50% FA were characterized by lower densities, which is related to the delayed hydration process of these binders and to the higher pore and air void content determined by the computed microtomography method shown in Fig. 8. The analysis of the porosity of the produced composites and the values of the total void content shown in Table 4 confirm the higher proportion of pores in the systems with FA. A reduction in the porosity of the composites was only obtained for the Portland cement with TiO_2_ (1Ti) and CaCO_3_ (1CC) and for the ternary system 2FA25CC, which confirms that the introduction of both additives accelerates the hydration of the binder and leads to a densification and refinement of the composite microstructure due to a more efficient nucleation. It was observed that for the vast majority of the systems analyzed, mechanical strength, density, photocatalytic degradation efficiency and global warming potential decrease with increasing porosity. The presented distributions of pore diameters and volumes also indicate that the configurations characterized by the highest total porosities (mainly systems containing 50% FA) are associated with a greater number of medium and large pores, compared to the systems containing 0 or 25% FA. These analyses are also confirmed by the selected scanning electron micrographs of the composite microstructure shown in Fig. 9. A comparison of the two images (sample 1 and 1Ti) shows a well densified microstructure of the cement slurry, characterized by a homogeneous structure and low pore content. In the subsequent images with 25 and 50% FA (2FA25 and 3FA50) respectively, there is a clear increase in the heterogeneity of the mortar, manifested by a higher pore content and a high content of unreacted fly ash grains. This also demonstrates the slower hydration of the cementitious binder and is in agreement with the other analyses.

The influence of nanometric TiO_2_ up to 4 wt% in the presence of large amounts of fly ash (20 wt% at the expense of cement) on the hydration process of the cement binder and the mechanical properties was studied by Ma et al.^37^. They showed that the optimum amount of TiO_2_ was 3 wt% and that this nano-additive had a positive effect on the early strength by accelerating the hydration process, whereas, as in our case, it reduced the workability of the composites and worsened the mechanical properties in the long term. They also pointed out that the negative effect of TiO_2_ can be compensated by the presence of fly ash, which contributes to the strength of the cementitious slurry at later stages of curing through the pozzolanic reaction^31^.

**Table 4:** Density and total voids volume of analyzed samples as well as SEM images of representative samples.

Sample	Density of samples (g/cm^3^)			Total voids volume (%)
	After 7 days	After 21 days	After 90 days	
1	2.27	2.29	2.32	5.94
2FA25	2.25	2.25	2.31	6.22
3FA50	2.15	2.20	2.27	6.53
1Ti	2.33	2.34	2.37	5.41
2FA25-Ti	2.27	2.30	2.31	6.63
3FA50-Ti	2.22	2.25	2.26	8.06
1CC	2.27	2.28	2.34	5.48
2FA25-CC	2.25	2.23	2.31	4.75
3FA50-CC	2.21	2.18	2.24	5.95
1CC-Ti	2.31	2.32	2.37	6.31
2FA25-CC-Ti	2.29	2.27	2.31	6.19
3FA50-CC-Ti	2.22	2.24	2.28	7.59

### Photocatalytic activity

**Fig. 10:**
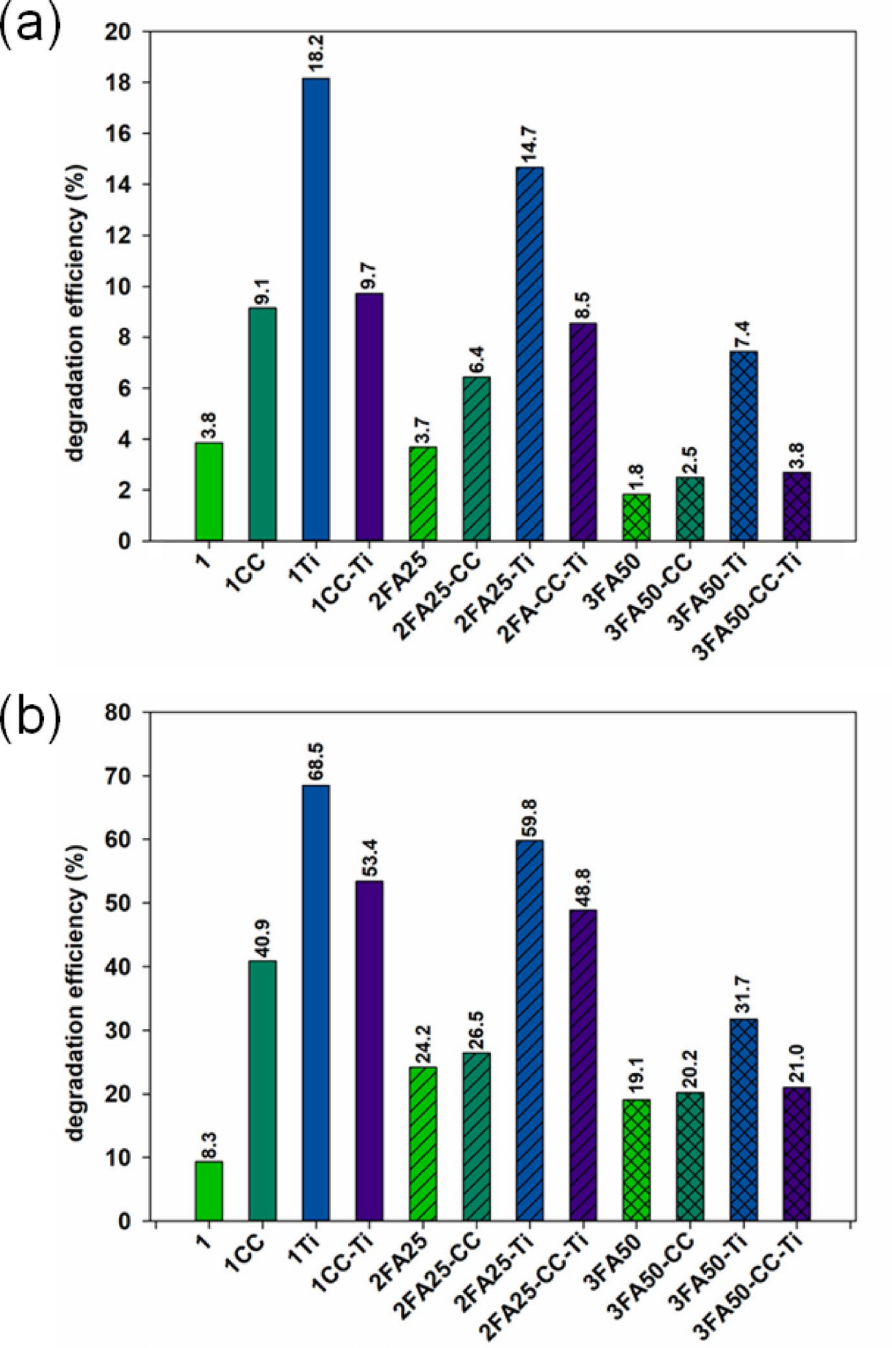
Results of the assessment of the photocatalytic properties of cement composites in terms of the efficiency of removing a model organic pollutant, namely a 10 mg/L phenol solution, after 6 h (**a**) and 24 h (**b**) of exposure to ultraviolet radiation.

The addition of nanometric titanium dioxide particles to cement mortars is expected to enable the production of materials with photocatalytic activity. Therefore, an assessment of the photocatalytic properties of cement composites was conducted, and the results are presented in Fig. 10. Based on the analysis of these results, it was concluded that the introduction of titanium dioxides into the cement matrix (1Ti sample), contributes to the production of cement composites with the best photocatalytic properties among the samples analyzed. For the 1Ti cement mortar, the phenol degradation efficiency was 18.2 and 68.5%, respectively, after 6 and 24 h of irradiation. In the case of adding calcium carbonate to the cement samples, there was also an improvement in phenol removal efficiency from the solution. For example, in the samples 1CC, the efficiency increased from 3.8 to 9.1% and from 8.3 to 40.9%, respectively, after 6 and 24 h of exposure. On the other hand, the addition of fly ash (2FA25 and 3FA50) resulted in lower efficiencies in the removal of the model organic pollutant, especially in the case of samples containing titanium dioxide as an addition component. For composites without the addition of TiO_2_ and calcium carbonate, the addition of fly ash resulted in higher phenol removal efficiencies after 24 h of exposure. The observed phenol removal in samples without the addition of titanium dioxide is most likely associated with the adsorption phenomenon on the composite surface rather than the photocatalytic decomposition of the pollutant, as the presence of a photocatalyst like TiO_2_ is required for the latter.

The obtained results are in line with existing literature. For instance, in the study by Baral et al.^39^. cement composites were manufactured in which ordinary Portland cement was replaced with 15% by weight of fly ash. Additionally, to impart photocatalytic properties, 2.5 and 5.0% by weight of titanium dioxide was introduced. It was observed that increasing the amount of introduced TiO_2_ above 2.5% by weight does not significantly enhance the efficiency of the NO_x_ removal reaction. However, replacing 15% by weight of cement with fly ash led to poorer photocatalytic properties^38^ similar to our findings. In the literature, this has been attributed to the fact that in cement composites with added fly ash, regions with lower pH are formed in the pores, which reduces the photocatalytic activity of titanium dioxide nanoparticles^38,39^. Conversely, in the study by King et al.^40^ introducing an appropriate amount of fly ash into the mortar, in their case, 15% by weight relative to the mass of the binder used, resulted in composites exhibiting the highest efficiency in photocatalytic degradation of NO_x_ after carbonation. This is attributed to the fact that fly ash reduces the amount of available CH (calcium hydroxide) on the composite surface capable of reacting with CO_2_, thereby minimizing the adverse effects of carbonation on photocatalytic properties. Consequently, composites containing TiO_2_ can maintain their photocatalytic properties over an extended period^35^. Regarding the influence of calcium carbonate on the photocatalytic properties of cement composites, the literature suggests that the presence of a positive effect on the hydration time carbonate in the mortar enhances the photocatalytic reaction of NO_x_ oxidation. This occurs because calcium carbonate reacts with the products of such a reaction, specifically nitrate(V) ions, resulting in the formation of calcium nitrates(V), which can be easily removed from the photocatalyst’s surface^41,42^. In the work of Kim et al.^43^. it was discovered that the formation of calcium carbonate on the sample’s surface due to carbonation covers the titanium dioxide particles. This leads to a significant reduction in the amount of TiO_2_ capable of being exposed to ultraviolet radiation, thus deteriorating the photocatalytic properties^43^. The shielding effect of CaCO_3_ in relation to titanium dioxide in cementitious matrix was also reported in the works of Diamanti et al.^44^. and Wang et al.^45^. This finding was confirmed in our research – the photocatalytic performance of sample 1CC-Ti was lower than for 1Ti.

### Life cycle assessment

**Fig. 11:**
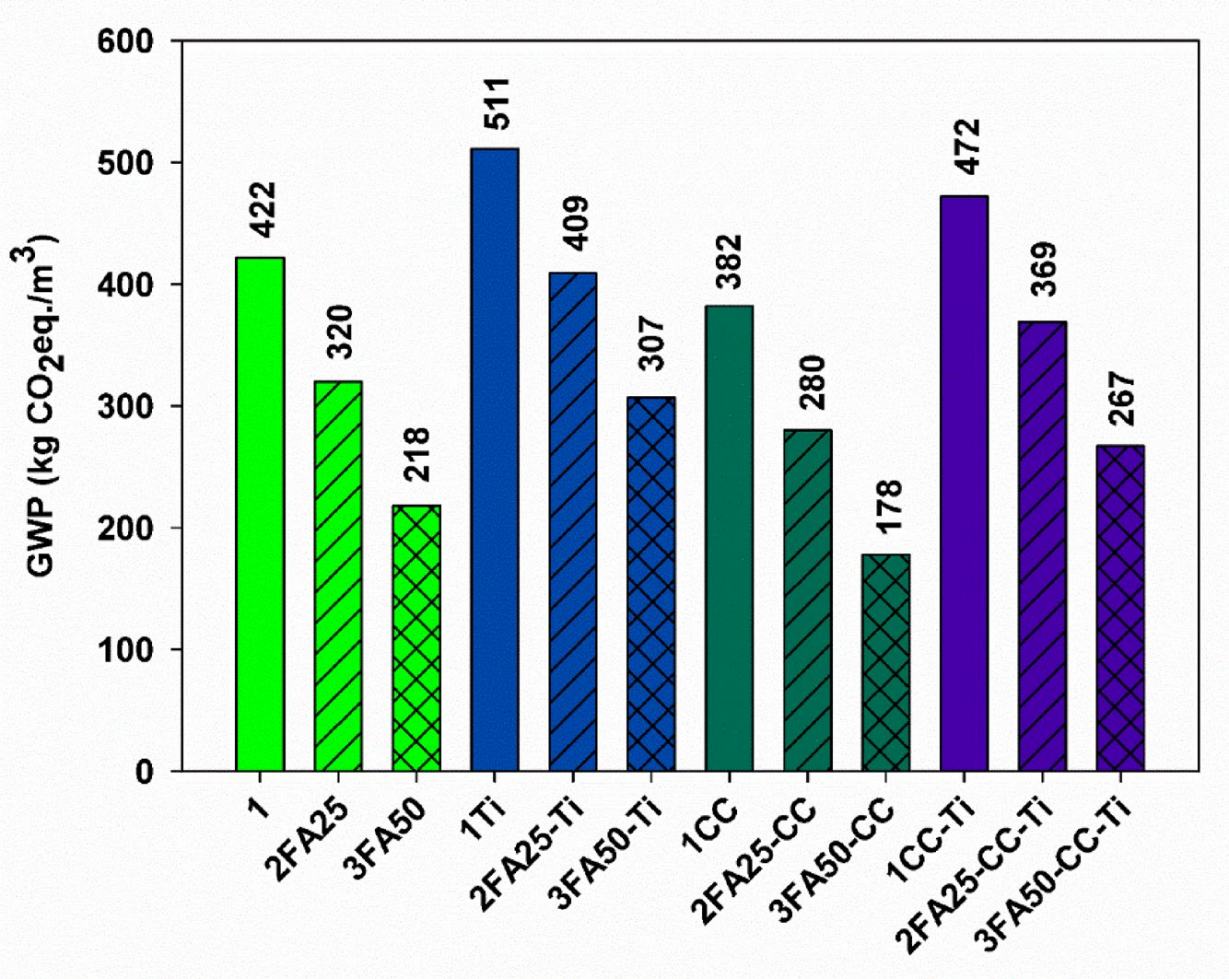
Global warming potential of prepared mixes.

The results of the LCA analyses carried out on the mixes produced are shown in Fig. 11. The first three bars in the graph show the standard mortar (1 sample) and the substitution of 25 and 50% fly ash in place of clinker (2FA25 and 3FA50), respectively. It is noticeable that there is a significant (48%) decrease in global warming potential GWP as the FA content increases to 50%, indicating that the clinker is responsible for almost all of the GWP of the mixtures without other additives, and that reducing its content by 50% reduces the GWP of the mixture by as much as 48%, which is possible because FA has a very low GWP. The next three bars show the original mixes in which the amount of clinker was further reduced by adding TiO_2_ at 5% of the original cement weight. This substitution increased the GWP by 21, 28 and 40% for mixes containing 0 (1Ti), 25 (2FA25-Ti) and 50% FA (3FA50-Ti) respectively. However, it is worth noting that mixes containing TiO_2_ and at least 25% FA (2FA25-Ti and 3FA50-Ti) had a lower GWP than a typical 100% clinker mortar and that their GWP decreased with increasing FA content. The next three bars show the GWP for mixes in which, in addition to FA, 10% of the original cement weight was replaced by calcium carbonate. The addition of CC reduced the GWP of all mixes by 10, 12 and 18% for FA contents of 0 (1CC), 25 (2FA25-CC) and 50% (3FA50-CC) respectively. The mix with 10% CC substitution had the lowest GWP and the mix with 10% CC, 50% FA and 40% clinker as binder (3FA50-CC sample) had the most favorable GWP. CC has a beneficial effect in reducing the GWP of the mixes. The last 3 bars show mixes containing both 10% CC and 5% TiO_2_ as binders and 0 (1CC-Ti), 25 (2FA25-CC-Ti) and 50% FA (3FA50-CC-Ti) respectively. Once again, it can be seen that the GWP decreases with increasing FA content, but unfortunately these blends have a higher GWP than those containing only FA and/or CC. It is worth noting that in this case the increase for blends containing only 0, 25 and 50% FA is lower than for blends containing only TiO_2_, being 12, 15 and 22% respectively. Furthermore, the mixes containing 25% FA, 5% TiO_2_ and 10% CC (2FA25-CC-Ti) had a lower GWP than the standard mix containing only clinker as a binder. The GWP of the 2FA25-CC-Ti and 3FA50-CC-Ti mixes was 13 and 37% lower, respectively, than that of a mix containing 100% Portland cement. Other researchers have shown that the use of SCMs such as MSWI FA (municipal solid waste incineration fly ash), FA or GGBS reduced the GWP of cement composites by up to 55%, while an increase in SCM content resulted in a decrease in the GWP of cement composites^46–48^.

The analyses carried out showed that, despite the negative effect of TiO_2_ on the GWP of the prepared blends, it is possible to modify the composition of the blends in such a way as to obtain satisfactory mechanical and photocatalytic parameters, while reducing the GWP of the blend based only on clinker as a binder. The GWP for nano-TiO_2_ and recycled aggregate (RA) concretes was determined by Moro et al.^49^. They showed that the presence of nano-TiO_2_ in concretes is associated with an increase in GWP values, the higher the nano content, the higher the GWP. They also found that replacing part of the natural aggregate with recycled aggregate counteracts this effect, leading to a significant reduction in the GWP of concrete mixes. They also showed that concrete made from 50% RA containing 5% FA and 0.3% TiO_2_ removed almost 70% of the pollutants in the form of NO_x_ due to the photocatalytic properties of TiO_2_. A positive effect of 69% GWP reduction was shown by Batuecas et al.^50^ by adding 2 wt% CaCO_3_ to Portland cement.

## Conclusions

An extensive analysis of the research results, together with a discussion of the existing literature data, allowed the following conclusions to be formulated:

1. The presence of titanium dioxide at a level of 5% by weight of cement in two-, three- and four-component systems resulted in a decrease in the slump of the cement composites produced, while it caused an acceleration of the heat of hydration released during the first 20 h of setting, induced by a more efficient nucleation.
2. In the case of pure Portland cement, a positive effect of nano-TiO_2_ on the improvement of the mechanical properties of the cement composites was observed, with an increase in the early compressive strength after 21 days of setting and a thickening of the cement slurry structure associated with the refinement of the porous structure. Favorable changes in strength were also observed for the addition of 10% calcium carbonate and for a ternary system with 25% FA and 10% CaCO_3_.
3. The presence of fly ash at high levels of 25 and 50% resulted in a reduction of the heat of hydration released, below 270 and 200 J/g respectively for all the systems studied, which in turn contributed to a slower rate of strength development throughout the study period. Nevertheless, favorable values for the strength parameters were obtained for the two, three- and four-component systems with 25% FA content. At this level, FA also improved the slump of the compounds produced.
4. The addition of titanium dioxide to pure Portland cement resulted in the best photocatalytic properties of the composite, with phenol degradation efficiencies of 18.2 and 68.5% observed after 6 and 24 h of irradiation, respectively. A positive photocatalytic effect was also observed for the two-component system with 10% CaCO_3_ content; in this case, the phenol degradation efficiency increased from 3.8 to 9.1% and from 8.3 to 40.9% after 6 and 24 h of exposure, respectively. Unfortunately, the presence of fly ash in the composites resulted in a decrease in photocatalytic efficiency, and this trend increased with increasing amounts of FA in all the composites tested.
5. In contrast, the addition of large amounts of fly ash and calcium carbonate in two-, three- and four-component systems resulted in lower GWPs, and the presence of both additives offset the negative effects of producing composites with nano-TiO_2_. This resulted in green composites with self-cleaning properties for the construction industry.

In summary, the need to reduce the carbon footprint of modern cements is leading to the increasing replacement of cement clinker with additives. Unfortunately, the replacement of clinker with increasing amounts of fly ash and calcium carbonate makes the cement matrix more porous and slows down the hydration process. Nanometric titanium dioxide can both counteract this phenomenon and impart photocatalytic properties to the cement composite, which have a positive effect on the degradation of pollutants and contribute to a more durable cement matrix. Unfortunately, the photocatalytic properties were masked with increasing amounts of FA. In the future, the durability of the proposed systems should be tested over a longer period of use, and research should be carried out to optimize the titanium dioxide content and increase its photocatalytic activity. This will contribute to the development of cement composites with lower GWP and reduced production costs.

## Data Availability

Data will be made available on request. Corresponding author Łukasz Klapiszewski is responsible for all data (lukasz.klapiszewski@put.poznan.pl).
